# Repetitive Direct Comparison Method for Odor Sensing

**DOI:** 10.3390/bios13030368

**Published:** 2023-03-10

**Authors:** Gaku Imamura, Kosuke Minami, Genki Yoshikawa

**Affiliations:** 1 International Center for Materials Nanoarchitectonics (MANA), National Institute for Materials Science (NIMS), 1-1 Namiki, Tsukuba 305-0044, Japan; 2 Graduate School of Information Science and Technology, Osaka University, 1-2 Yamadaoka, Suita 565-0871, Japan; 3 Research Center for Functional Materials, National Institute for Materials Science (NIMS), 1-1 Namiki, Tsukuba 305-0044, Japan; 4 Materials Science and Engineering, Graduate School of Pure and Applied Science, University of Tsukuba, 1-1-1 Tennodai, Tsukuba 305-8571, Japan

**Keywords:** gas sensor, olfactory sensor, signal processing, membrane-type surface stress sensor

## Abstract

Olfactory sensors are one of the most anticipated applications of gas sensors. To distinguish odors—complex mixtures of gas species, it is necessary to extract sensor responses originating from the target odors. However, the responses of gas sensors tend to be affected by interfering gases with much higher concentrations than target odor molecules. To realize practical applications of olfactory sensors, extracting minute sensor responses of odors from major interfering gases is required. In this study, we propose a repetitive direct comparison (rDC) method, which can highlight the difference in odors by alternately injecting the two target odors into a gas sensor. We verified the feasibility of the rDC method on chocolates with two different flavors by using a sensor system based on membrane-type surface stress sensors (MSS). The odors of the chocolates were measured by the rDC method, and the signal-to-noise ratios (S/N) of the measurements were evaluated. The results showed that the rDC method achieved improved S/N compared to a typical measurement. The result also indicates that sensing signals could be enhanced for a specific combination of receptor materials of MSS and target odors.

## 1. Introduction

Olfaction plays an important role in judging the quality of food; for example, one can detect spoilage of foods and estimate the ripeness of fruit with his/her nose. In the food industry, the flavor is one of the most important factors of products [1]. Food companies make great efforts to improve the flavor of their products, while logistics companies and retailers maintain and control the food quality, including flavors, during transportation and storage. To automate the quality control of flavors, olfactory sensors—also known as electronic noses (e-noses), artificial olfaction, or machine olfaction—have been attracting much attention [2]. Numerous studies utilizing olfactory sensors on food products have been reported, such as the identification of flavors and the detection of ripening, spoilage, and adulteration [3,4]. Despite such studies, the number of practical applications of olfactory sensors in the food industry is still limited. This is due to technical problems of olfactory sensors stemming from the complex nature of odor.

**Figure 1:**
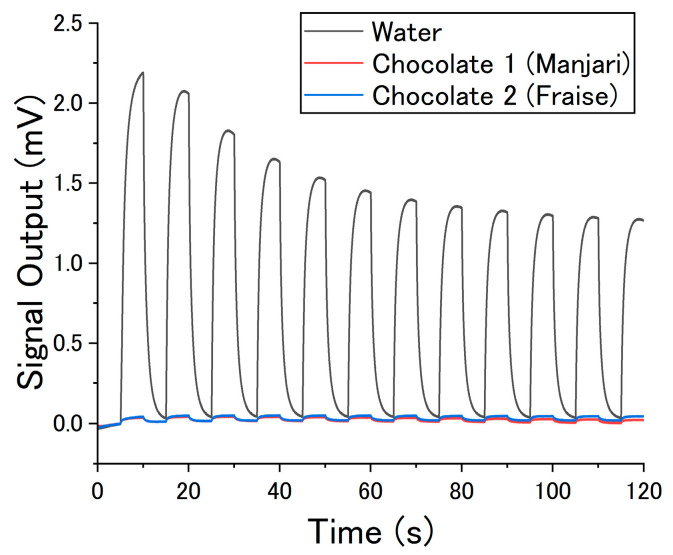
Sensing outputs of the measurements with the PPPO-coated MSS. Vapors of ultrapure water and two chocolates (“Manjari” and “Fraise” flavors) were used as samples. The sample vapors and the carrier gas (deodorized and dehumidified air) were alternately injected into the MSS with an interval of 5 s. The baselines are corrected so that the signal output at 5 s is 0 mV. The measurement protocol follows the normal measurement, which is explained in Section 2.

As an olfactory sensor is a gas sensor system that mimics human odor perception, an olfactory sensor should consist of two main parts: detection of odor molecules and identification of an odor [5]. In the mammalian olfactory mechanism, odor molecules inhaled through the nose are detected by olfactory receptors in the nasal epithelium. There are approximately 400 types of olfactory receptors in humans [6]. Each olfactory receptor has a different affinity for odor molecules and transduces the chemical interactions into neural signals. The signals are then transmitted to the olfactory bulb, followed by odor identification at the limbic system. To realize practical olfactory sensors, therefore, it is necessary to improve both odor detection and identification [7,8]. Recent dramatic progress in artificial intelligence has led to various studies utilizing data science approaches in olfactory sensors [9]. Although such approaches are effective in identifying odors from sensor data, the data should be collected with an appropriate measurement protocol so that the data contain information about the odors. In many cases, the concentration of target odor molecules (mostly volatile organic compounds, VOCs) is quite low, on the order of parts per million (ppm; 10^−6^) or less. Sensing signals for such low-concentration odor molecules are hindered by interfering gases that exist at high concentrations. Figure 1 shows an example of gas sensor measurements of water and chocolates with different flavors. The sample vapors and carrier gas (cleaned air) were alternately injected into a gas sensor (a membrane-type surface stress sensor (MSS) coated with poly(2,6-diphenyl-p-phenylene oxide) (PPPO)). The details of the measurements are explained in Section 2. As observed in this example, the sensing output for water vapor, which is a typical interfering gas, is much higher than those for the target odors in many cases; odor molecules are present at very low concentrations and contribute little to the sensor response, while background or interfering gases strongly affect the sensor response. It should be noted that the sensor responses reflect the composition of the gases, but the responses are not necessarily related to human olfaction. It is inferred from Figure 1 that both eliminating the effect of interfering gases and detecting low-concentration gases with a high signal-to-noise ratio (S/N) are essential in odor sensing.

To make reliable olfactory sensors that are applicable in the food industry, it is necessary to design a sensing system that is robust to interfering gases in order to detect the slight difference between odors. One such approach is the elimination of interfering gases by filters or traps [10]. Although this approach works well in some cases [11], such filters or traps may eliminate target molecules as well as the interfering gases. The use of a preconcentrator and micro-gas chromatography are other approaches for detecting low-concentration VOCs from interfering gases [12,13,14]. This approach, however, often requires a temperature control unit in the measurement system and a long measurement time, resulting in a complicated measurement protocol.

Another issue in realizing reliable olfactory sensors is increasing the S/N of the measurement system. One of the most common methods for improving S/N is signal averaging, which is widely used in many types of sensor applications. However, such an approach has rarely been applied to gas sensors because it has been difficult to obtain reproducible sensor responses from repeated measurements. Gas sensors, including metal oxide sensors, quartz crystal microbalance sensors, and chemiresistive sensors, absorb or adsorb target gases on their sensing units. These types of sensors often suffer from the effect of residual gases because it takes some time for the absorbed/adsorbed gases to desorb from the sensing units. To obtain reproducible responses with such sensors, the residual gases must be completely eliminated by purging with carrier gas at each repetition, which takes too long for practical use. One exception to this is optical gas sensors, which detect and analyze light passing through a cell filled with a sample gas [15,16,17,18,19]. As the probe light does not usually have a chemical interaction with gas samples, the signal averaging can be performed with a repetitive measurement over the light exposure time. In addition, as the cell can be easily cleaned by carrier gas purging, the sensing response is unlikely to be affected by residual gas. In contrast to this exceptional case in optical gas sensors, improving S/N by signal averaging remains a challenging task in chemical gas sensor measurements.

In this study, we have developed a repetitive direct comparison (rDC) method that can highlight the difference between odors by alternately injecting target odors into a gas sensor. The key feature of this measurement method is that the effects of common interfering gases are canceled, and the sensor responses based on the difference in VOCs of each sample can be obtained. This is the most distinctive feature of the rDC method from typical odor measurements where sensing responses are separately obtained for each sample. As such, sensor responses are weak due to the low concentration of VOCs—typically less than ppm order. We repeated the cycles of odor injections and averaged the sensing outputs to improve the S/N. Since it has already been demonstrated with MSS that the reproducible sensing outputs can be obtained after some repetitions even though the receptor layer contains residual gases [20,21], S/N can be improved by signal averaging, which has rarely been applied to gas sensor measurements.

## 2. Materials and Methods

### 2.1. Measurement Setup

**Figure 2:**
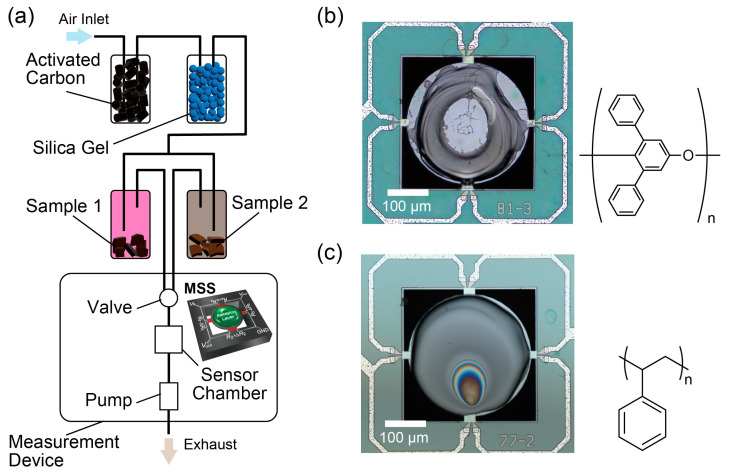
(**a**) A schematic illustration of the measurement setup for the rDC method. In this study, MSS was used as a gas sensor. (**b**,**c**) Optical microscope images of MSS coated with (**b**) PPPO and (**c**) PS. The molecular structures of PPPO and PS are shown on the right of the microscope images.

Figure 2a shows the measurement setup for the rDC method. Two samples were set in the vials, and their headspace gases were injected into the sensors. Unlike typical odor measurement methods where an odor of a sample and a carrier gas are alternately delivered to a gas sensor [22,23], the odors of the two samples are alternately injected in this method. The rDC method is effective for samples mainly composed of the same base components but have slightly different odors because the sensing responses highlight the difference in odors. In this study, two kinds of chocolates were used as samples. For comparison, we also measured the odors of the chocolates with a typical measurement protocol in which the odor and the cleaned air were alternately injected (hereafter, denoted as “normal measurements”). Such measurements were performed by setting the chocolates in the Sample 1 vial and emptying the Sample 2 vial. The normal measurement of ultrapure water was performed with the same configuration. The outer air was deodorized and dehumidified for the measurements by passing through the glass vials filled with activated carbon pellets and silica gel beads, as shown in Figure 2a. The cleaned air was flown to the vials for 5 min before the measurements to fill the vials with the cleaned air. By using the cleaned air as a carrier gas, the headspace gases of the two vials containing samples were delivered to a sensor chamber in which MSS (purchased from NanoWorld AG) coated with two different polymers (i.e., PPPO and polystyrene (PS)) were set. The flow rate was controlled by a pump and set at 30 mL/min. All the measurements were conducted at 25 °C. The repetitive measurements were carried out by switching the flow paths with a valve placed in front of the sensor. Odors of Sample 1 and Sample 2 were alternately injected with an interval of 5 s. The repetitions of the sample odor injections started with Sample 2. The total measurement duration was set at 120 s. To stabilize the device, measurements without samples were performed for several hours prior to the experiments.

We also conducted normal measurements on Manjari and Fraise chocolates. A measurement with the rDC method was performed by setting Manjari and Fraise chocolates in Sample 1 and Sample 2 vials, respectively. This set of measurements was performed 6 times, and the performance was evaluated statistically. As the sensor chamber contains both the PPPO- and the PS-coated MSS, the sensing outputs for the two MSS could be obtained simultaneously.

### 2.2. Odor Samples

Chocolates with different flavors, i.e., “Fraise” and “Manjari” flavors (purchased from Valrhona), were used as odor samples. Although the flavor of chocolate consists of several hundred VOCs [24], the compositions of the vapors from the two kinds of chocolates are considered to be similar. VOCs from the base material—cacao, milk, and butter—are dominant, while the flavors are additional components. Fraise and Manjari chocolates have strawberry and vanilla flavors, of which representative components are furaneol and vanillin, respectively [25]. Thus, it is expected that only a slight difference originating from the flavors could be seen in sensing responses with the rDC method.

### 2.3. Membrane-Type Surface Stress Sensors (MSS)

Nanomechanical sensors are sensors that detect changes in mechanical properties such as volume, strain, and stress. Since first demonstrated with micro-cantilevers in 1994 [26,27], nanomechanical sensors have been shown as a great sensing platform [28,29,30]. MSS, which was developed in 2011 by Yoshikawa et al., is one of the nanomechanical sensors [31]. An MSS is a small gas sensor fabricated through a micro-electro-mechanical systems (MEMS) process [31]. With its distinctive structure (shown in Figure 2a), MSS efficiently detects surface stress caused by mechanical deformation of a receptor layer, resulting in high sensitivity (i.e., sub-ppm level gas detection) [32,33]. The receptor layer coated on the sensing membrane absorbs gaseous molecules to expand. The expansion results in the deformation of the sensing membrane and induces surface stress, which is electrically read by the four piezoresistors. As the piezoresistors compose the Wheatstone bridge circuit, the sensing output ($V_{\mathrm{out}}$) is obtained via the following formula [31]:

(1) $$V_{\mathrm{out}}=\frac{V_{B}}{4}\left(\frac{\Delta R_{1}}{R_{1}}-\frac{\Delta R_{2}}{R_{2}}+\frac{\Delta R_{3}}{R_{3}}-\frac{\Delta R_{4}}{R_{4}}\right),$$

where $V_{B}$ is the bridge voltage applied to the circuit. $R_{1}$, $R_{2}$, $R_{3}$, and $R_{4}$ are the resistance of the piezoresistors, and $\Delta R_{1}$, $\Delta R_{2}$, $\Delta R_{3}$, and $\Delta R_{4}$ are the changes in the resistance of the piezoresistors. As receptor layers, PPPO and PS were used in this study. PPPO, which is also known as Tenax^®^, is typically used as a sorbent for low-concentration VOCs. The superior absorption ability of PPPO is also preferable as a receptor material of nanomechanical sensors, which we confirmed in our previous study [21]. PS has been widely used in nanomechanical sensors, and its characteristics as a receptor material are well-studied [20,34]. The molecular structures of PPPO and PS are shown in Figure 2b,c. These two polymers are hydrophobic properties, which are thought to be preferable for detecting odor molecules [22]. These polymers were dissolved in 1,1,2,2-tetrachloroethane and coated on MSS with an inkjet spotter. Optical microscope images of the PPPO- and PS-coated MSS are shown in Figure 2b,c. Even though the receptor materials are not uniformly coated, the sensing outputs of MSS are robust to the topography of the receptor layer compared to cantilever-type sensors [35]. Sensing signals of MSS were read with $V_{B}=-1.0$ [V] at a sampling frequency of 100 Hz. More details of MSS are described in Refs. [31,36].

### 2.4. Signal Processing

**Figure 3:**
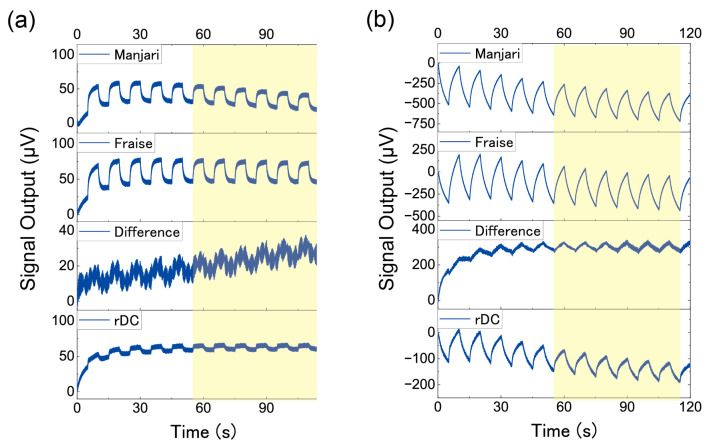
Whole sensing outputs of the measurements with (**a**) the PPPO-coated MSS and (**b**) the PS-coated MSS. Baselines are corrected at 0 s. Yellow-shaded areas are used for averaging.

Time-series data of sensing outputs were obtained from the measurements. The sensing outputs between 55 s and 115 s were used for analysis (yellow shaded area in Figure 3). Averaging of the signal outputs was performed by the following process: the sensing outputs were divided into 6 parts according to the repetitions. The baseline was corrected for each part so that the sensing output at the start of Sample 1 injection becomes 0. After the baseline correction, the sensing outputs of the 6 parts were averaged.

The noise levels were evaluated with the sensing outputs of the last 1 s of the repetition. As the sensing outputs can be considered linear in this period, the sensing outputs were fitted with straight lines. The noise levels were calculated from the residuals; the noise level was defined by the root mean square (RMS) of the residuals.

Signal intensity is defined as the difference between the initial and peak sensing outputs in a cycle, the difference between the signal outputs at 0 s and 5 s.

## 3. Results

Figure 3 shows the whole sensing outputs from one set of the six measurements: the normal measurements of Manjari and Fraise chocolates, the difference between Manjari and Fraise chocolates, and the measurements by the rDC method of Manjari and Fraise chocolates. The baselines were corrected so that the sensing outputs become 0 μV at 0 s. The difference between Manjari and Fraise was calculated by subtracting the Manjari sensing outputs from the Fraise sensing outputs. Figure 3a,b show the results of the MSS coated with PPPO and PS, respectively. The results of normal measurements of Manjari and Fraise shown in Figure 3a are also shown in Figure 1. Clear signal peaks can be seen for Manjari and Fraise chocolates; signal peaks and valleys correspond to the injection of the odors and the carrier gas (i.e., cleaned air), respectively. As explained above, these signals are basically induced by the difference between chocolates and carrier gases, reflecting almost all components in each chocolate sample, including major base components. In contrast, lower signal peaks compared to the normal measurements of Manjari and Fraise are observed for the rDC method reflecting the slight difference in odors. For any measurement, the signal shapes for a single cycle of odor injections gradually change for the first few repetitions because of the sensing mechanism of MSS; sensing outputs reflect the gas diffusion process in the receptor layer and the changes in viscoelastic properties of the receptor materials [20,37,38]. After some repetitions, the sensing outputs reach a steady state; for each repetition, almost the same signal appears. The baseline shifted slightly even after the sensing outputs reached the steady state, which may be due to the temperature shift associated with the thermal heating of the circuit. Such sensing outputs can be averaged to improve S/N.

**Figure 4:**
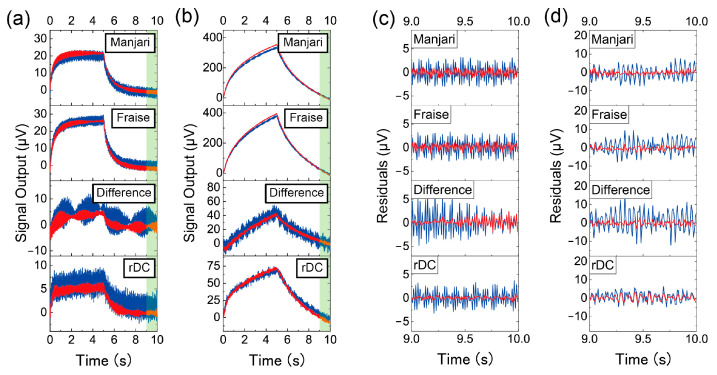
Raw (blue) and averaged (red) sensing outputs from (**a**) the PPPO-coated MSS and (**b**) the PS-coated MSS. Residuals of sensing outputs for the linear fit over the time period of 9.0 to 10.0 s (green-shaded area in (**a**) and (**b**)) for (**c**) the PPPO-coated MSS and (**d**) the PS-coated MSS. Blue and red lines indicate raw and averaged sensing outputs, respectively.

The effect of the averaging is shown in Figure 4. Blue and red lines represent the raw and averaged sensing outputs, respectively. The results of the MSS coated with PPPO and PS correspond to Figure 4a,b, respectively. Raw sensing outputs shown in Figure 4a,b were trimmed from the last cycle in the yellow shaded in Figure 3. It is clear from Figure 4a,b that the noise levels were reduced after the averaging. Figure 4c,d are the residual plots for the PPPO- and PS-coated MSS, respectively, representing the difference between the experimental values and the expected values from the linear fit. This analysis was performed in the range between 9.0 s and 10.0 s (green shaded area in Figure 4a,b). It is also clear from Figure 4c,d that the noise level was reduced by the signal averaging for each case. As can be seen from Figure 4c,d, the noise level of the difference between Fraise and Manjari is higher than that of other measurements due to the propagation of errors by signal processing of two data with noise.

**Figure 5:**
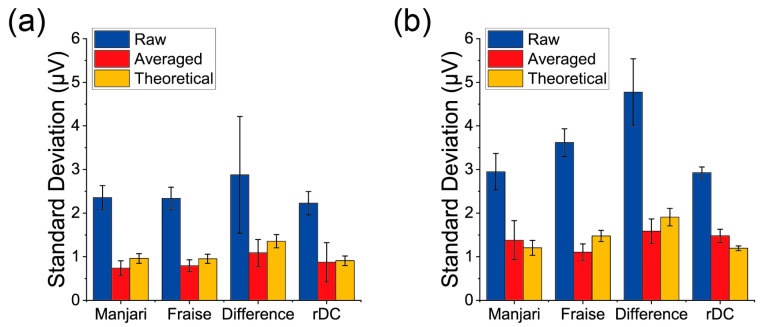
Summary of the noise level of each measurement with (**a**) the PPPO-coated MSS and (**b**) the PS-coated MSS. Error bars represent the standard deviations.

The noise levels for the PPPO-coated MSS and the PS-coated MSS are summarized in Figure 5a,b, respectively. The bars and error bars represent the mean and standard deviation of the noise level evaluated from the six measurements. Noise reduction was confirmed for all the measurements after averaging. Theoretical values are also shown in Figure 5 as references. If the noise is considered to have a Gaussian distribution, the noise level is inversely proportional to the square root of N. Thus, theoretical values for normal measurements on Manjari and Fraise chocolates and the rDC method are calculated by dividing the noise levels of the raw sensing outputs with $\sqrt{N}$, where N is the number of repetitions. In this study, N=6 corresponds to the number of repetitions in the yellow-shaded areas in Figure 3. The theoretical values for the difference between Manjari and Fraise were calculated by the following form:

(2) $$\sqrt{\sigma_{\mathrm{th},\mathrm{Manjari}}^{2}+\sigma_{\mathrm{th},\mathrm{Fraise}}^{2}},$$

where $\sigma_{\mathrm{th},\mathrm{Manjari}}^{2}$ and $\sigma_{\mathrm{th},\mathrm{Fraise}}^{2}$ are the theoretical noise levels (i.e., RMS) for the normal measurements of Manjari and Fraise chocolates, respectively. Although noise levels were reduced by averaging, deviations from the theoretical values were observed in some cases: the normal measurement of Fraise and the rDC method with the PS-coated MSS. Moreover, undulations in noises can be seen for some measurements in Figure 4a,b. The deviation from the theoretical expectation might be due to such noise that does not follow a Gaussian distribution. Such noise components, which can originate from electronic noises from the circuits, including pumps and valves, could be canceled or enhanced by averaging.

## 4. Discussion

**Figure 6:**
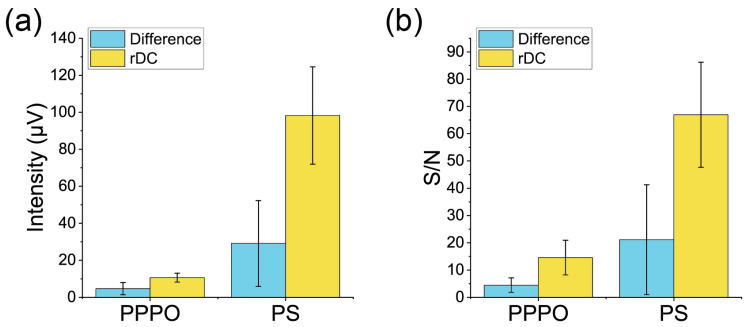
Summary of (**a**) the intensity and (**b**) S/N. Error bars represent the standard deviations.

The signal intensities, noise levels after averaging (from Figure 5), and S/N are summarized in Figure 6 and Table 1. S/N was calculated for each measurement, and the mean and standard deviation were evaluated from the S/N. In the case of the PPPO-coated MSS, the signal intensity is slightly higher for the rDC method than the difference of the normal measurements, while the noise level is almost the same for both methods. A comparison of the S/N shows that the rDC method achieved a slightly higher S/N. An interesting feature can be seen for the PS-coated MSS. The rDC method achieved significantly higher signal intensity than the normal measurement. This enhancement effect might be due to the absorption/desorption dynamics of VOCs at the receptor layer. As the receptor layer is not purged with a carrier gas for the rDC method, common VOCs between the two samples remain in the receptor layer during the measurement. Such remaining VOCs may interact with the different components of the odors and cause nonlinear expansion of the receptor layer, leading to enhanced signal intensity. The result implies that a specific selection of receptor material can heighten the sensing signals originating from the difference in odors. However, further investigation is needed to clarify the mechanism of signal enhancement.

Although signal averaging is a common technique in many types of sensor measurements, it has rarely been applied to gas sensor measurements, except for optical gas sensors [15,16,17,18,19]. The reason is that the same signal outputs could hardly be obtained with gas sensors because of residual gases in their sensing units. In this study, we approached this problem by allowing the sensing responses to reach a steady state by repeating the cycle of sample gas injection and carrier gas purging. This approach has made it possible to obtain reproducible sensing outputs for each repetition without fully cleaning the sensing units. The theoretical background for the sensing responses of MSS in repetitive measurements has been investigated in Refs. [20,21]. This approach is not limited to MSS and can be applied to other types of gas sensors.

Besides averaging the sensing outputs after repetitions of odor injections, noise reduction techniques such as low-pass filters and moving average filters are typically used for reducing the noise. While such methods can efficiently reduce high-frequency noise, the methods may also attenuate the rapid sensor response, which contains information on the interaction between the sensing element and the gas molecules. The responses of MSS, for example, reflect the dynamics of gas sorption and viscoelastic stress relaxation of the receptor layer, with time constants varying typically between 0.1 to 100 s [20,39]. As the rapid sensor response with a short time constant is important in odor discrimination and identification from the viewpoint of feature extraction for machine learning [22,40], noise reduction methods that attenuate high-frequency components should be applied to the sensing outputs after careful parameter setting (e.g., cutoff frequency and window width).

## 5. Conclusions

In conclusion, we propose a measurement method in which the difference in odors of samples is highlighted. In this repetitive direct comparison (rDC) method, the odors of samples are alternately injected into a sensor without carrier gas purging. The cycle of the odor injections is repeated to obtain multiple sensing responses for averaging the outputs. As a demonstration, we measured the odors of chocolates by the rDC method and detected the sensor responses originating from the difference in flavors with higher S/N than a typical measurement protocol. By utilizing the rDC method, not only the common components from the base material but also the changes in the experimental conditions (e.g., temperature, humidity, and VOCs in the air) could be canceled, leading to applications such as odor quality control and the detection of defective products. If a standard product is available, the quality of each product can be examined by the rDC method without being affected by the disturbance of the experimental conditions. Since this measurement method is advantageous for highlighting the slight difference in odor, it can be used for various purposes such as quality control of food, monitoring of indoor air quality, and classification of cosmetics, especially for measuring samples consisting of large amounts of interfering components with different odors (e.g., fruit-flavored carbonated drinks and room fragrances). In a product inspection, for example, one can examine the odor of a product by comparing the product to a reference product with the rDC method; the product passes the inspection if the signal intensity is within a permission range. We believe this method will become a key technology for odor sensing and contribute to the widespread use of odor sensors.

## 6. Patents

Japanese patent application No. 2022-074098.

## Figures and Tables

**Table 1:** Summary of the signal intensities, noise levels, and S/N.

Receptor Material	Measurement Type	Signal Intensity (μV)	Noise Level after Averaging (μV)	S/N
PPPO	rDC	10.6 ± 2.4	0.87 ± 0.45	14.6 ± 6.4
PPPO	Difference of the Normal Measurements	4.7 ± 3.3	1.09 ± 0.31	4.5 ± 2.7
PS	rDC	98 ± 26	1.48 ± 0.15	67 ± 19
PS	Difference of the Normal Measurements	29 ± 23	1.58 ± 0.28	21 ± 20

## Data Availability

Not applicable.

## References

[1] Kader A.A. (2008). Flavor quality of fruits and vegetables. J. Sci. Food Agric..

[2] Shi H., Zhang M., Adhikari B. (2018). Advances of electronic nose and its application in fresh foods: A review. Crit. Rev. Food Sci. Nutr..

[3] Jia W., Liang G., Jiang Z., Wang J. (2019). Advances in Electronic Nose Development for Application to Agricultural Products. Food Anal. Methods.

[4] Tan J., Xu J. (2020). Applications of electronic nose (e-nose) and electronic tongue (e-tongue) in food quality-related properties determination: A review. Artif. Intell. Agric..

[5] Su C.-Y., Menuz K., Carlson J.R. (2009). Olfactory Perception: Receptors, Cells, and Circuits. Cell.

[6] Niimura Y., Nei M. (2003). Evolution of olfactory receptor genes in the human genome. Proc. Natl. Acad. Sci. USA.

[7] Persaud K., Dodd G. (1982). Analysis of discrimination mechanisms in the mammalian olfactory system using a model nose. Nature.

[8] Persaud K.C. (2012). Biomimetic Olfactory Sensors. IEEE Sens. J..

[9] Ye Z., Liu Y., Li Q. (2021). Recent Progress in Smart Electronic Nose Technologies Enabled with Machine Learning Methods. Sensors.

[10] van den Broek J., Weber I.C., Güntner A.T., Pratsinis S.E. (2021). Highly selective gas sensing enabled by filters. Mater. Horiz..

[11] Jirayupat C., Nagashima K., Hosomi T., Takahashi T., Samransuksamer B., Hanai Y., Nakao A., Nakatani M., Liu J., Zhang G. (2022). Breath odor-based individual authentication by an artificial olfactory sensor system and machine learning. Chem. Commun..

[12] Reddy K., Guo Y., Liu J., Lee W., Khaing Oo M.K., Fan X. (2012). Rapid, sensitive, and multiplexed on-chip optical sensors for micro-gas chromatography. Lab A Chip.

[13] James F., Breuil P., Pijolat C., Camara M., Briand D., Bart A., Cozic R. (2014). Development of a MEMS Preconcentrator for Micro-gas Chromatography Analyses. Procedia Eng..

[14] Biswas P., Zhang C., Chen Y., Liu Z., Vaziri S., Zhou W., Sun Y. (2021). A Portable Micro-Gas Chromatography with Integrated Photonic Crystal Slab Sensors on Chip. Biosensors.

[15] Werle P., Mücke R., Slemr F. (1993). The limits of signal averaging in atmospheric trace-gas monitoring by tunable diode-laser absorption spectroscopy (TDLAS). Appl. Phys. B.

[16] Bauer R., Stewart G., Johnstone W., Boyd E., Lengden M. (2014). 3D-printed miniature gas cell for photoacoustic spectroscopy of trace gases. Opt. Lett..

[17] Li J., Deng H., Sun J., Yu B., Fischer H. (2016). Simultaneous atmospheric CO, N_2_O and H_2_O detection using a single quantum cascade laser sensor based on dual-spectroscopy techniques. Sens. Actuators B Chem..

[18] Khan A., Schaefer D., Tao L., Miller D.J., Sun K., Zondlo M.A., Harrison W.A., Roscoe B., Lary D.J. (2012). Low Power Greenhouse Gas Sensors for Unmanned Aerial Vehicles. Remote Sens..

[19] Langridge J.M., Ball S.M., Shillings A.J.L., Jones R.L. (2008). A broadband absorption spectrometer using light emitting diodes for ultrasensitive, in situ trace gas detection. Rev. Sci. Instrum..

[20] Minami K., Shiba K., Yoshikawa G. (2021). Sorption-induced static mode nanomechanical sensing with viscoelastic receptor layers for multistep injection-purge cycles. J. Appl. Phys..

[21] Inada K., Kojima H., Cho-Isoda Y., Tamura R., Imamura G., Minami K., Nemoto T., Yoshikawa G. (2021). Statistical Evaluation of Total Expiratory Breath Samples Collected throughout a Year: Reproducibility and Applicability toward Olfactory Sensor-Based Breath Diagnostics. Sensors.

[22] Shiba K., Tamura R., Imamura G., Yoshikawa G. (2017). Data-driven nanomechanical sensing: Specific information extraction from a complex system. Sci. Rep..

[23] Ngo H.T., Minami K., Imamura G., Shiba K., Yoshikawa G. (2018). Effects of Center Metals in Porphines on Nanomechanical Gas Sensing. Sensors.

[24] Aprotosoaie A.C., Luca S.V., Miron A. (2016). Flavor Chemistry of Cocoa and Cocoa Products—An Overview. Compr. Rev. Food Sci. Food Saf..

[25] Sen A., Schieberle P., Grosch W. (1991). Quantitative-determination of 2,5-dimethyl-4-hydroxy-3(2H)-furanone and its methyl-ether using a stable isotope-dilution assay. Food Sci. Technol.-Lebensm.-Wiss. Technol..

[26] Gimzewski J.K., Gerber C., Meyer E., Schlittler R.R. (1994). Observation of a chemical reaction using a micromechanical sensor. Chem. Phys. Lett..

[27] Thundat T., Warmack R.J., Chen G.Y., Allison D.P. (1994). Thermal and ambient-induced deflections of scanning force microscope cantilevers. Appl. Phys. Lett..

[28] Lang H.P., Hegner M., Gerber C. (2005). Cantilever array sensors. Mater. Today.

[29] Sader J.E. (2001). Surface stress induced deflections of cantilever plates with applications to the atomic force microscope: Rectangular plates. J. Appl. Phys..

[30] Watari M., Galbraith J., Lang H.-P., Sousa M., Hegner M., Gerber C., Horton M.A., McKendry R.A. (2007). Investigating the Molecular Mechanisms of In-Plane Mechanochemistry on Cantilever Arrays. J. Am. Chem. Soc..

[31] Yoshikawa G., Akiyama T., Gautsch S., Vettiger P., Rohrer H. (2011). Nanomechanical Membrane-type Surface Stress Sensor. Nano Lett..

[32] Osica I., Imamura G., Shiba K., Ji Q., Shrestha L.K., Hill J.P., Kurzydłowski K.J., Yoshikawa G., Ariga K. (2017). Highly Networked Capsular Silica–Porphyrin Hybrid Nanostructures as Efficient Materials for Acetone Vapor Sensing. ACS Appl. Mater. Interfaces.

[33] Imamura G., Minami K., Shiba K., Mistry K., Musselman K.P., Yavuz M., Yoshikawa G., Saiki K., Obata S. (2020). Graphene Oxide as a Sensing Material for Gas Detection Based on Nanomechanical Sensors in the Static Mode. Chemosensors.

[34] Baller M.K., Lang H.P., Fritz J., Gerber C., Gimzewski J.K., Drechsler U., Rothuizen H., Despont M., Vettiger P., Battiston F.M. (2000). A cantilever array-based artificial nose. Ultramicroscopy.

[35] Loizeau F., Akiyama T., Gautsch S., Vettiger P., Yoshikawa G., de Rooij N.F. (2015). Comparing membrane- and cantilever-based surface stress sensors for reproducibility. Sens. Actuator A Phys..

[36] Minami K., Imamura G., Tamura R., Shiba K., Yoshikawa G. (2022). Recent Advances in Nanomechanical Membrane-Type Surface Stress Sensors towards Artificial Olfaction. Biosensors.

[37] Wenzel M.J., Josse F., Heinrich S.M., Yaz E., Datskos P.G. (2008). Sorption-induced static bending of microcantilevers coated with viscoelastic material. J. Appl. Phys..

[38] Heinrich S.M., Wenzel M.J., Josse F., Dufour I. (2009). An analytical model for transient deformation of viscoelastically coated beams: Applications to static-mode microcantilever chemical sensors. J. Appl. Phys..

[39] Imamura G., Shiba K., Yoshikawa G., Washio T. (2018). Analysis of nanomechanical sensing signals; physical parameter estimation for gas identification. AIP Adv..

[40] Imamura G., Shiba K., Yoshikawa G., Washio T. (2019). Free-hand gas identification based on transfer function ratios without gas flow control. Sci. Rep..

